# A new paradigm in postoperative colorectal cancer surveillance: integrating advanced imaging and multi-omics

**DOI:** 10.3389/fphys.2025.1758385

**Published:** 2026-01-05

**Authors:** Dongxue Yu, Jiandong Tai

**Affiliations:** 1 The Third Affiliated Clinical Hospital of Changchun University of Chinese Medicine, Jilin, China; 2 Department of Colorectal and anal Surgery, General Surgery Center, First Hospital of Jilin University, Changchun, China; 3 Changchun University of Traditional Chinese Medicine, Jilin, China

**Keywords:** colorectal cancer, minimal residual disease (MRD), multi-omics, postoperative surveillance, radiomics

## Abstract

Up to one-third of patients with localized colorectal cancer (CRC) relapse after curative-intent resection, as conventional markers like carcinoembryonic antigen (CEA) and scheduled CT/MRI often fail to detect micro-metastatic disease early. Advanced imaging, particularly radiomics, and liquid biopsy with circulating tumor DNA (ctDNA) are emerging as complementary tools to address this challenge. Radiomics extracts high-throughput image features to quantify risk and track response, with reported AUCs often ranging from 0.70 to 0.85. Concurrently, ctDNA has proven to be the strongest postoperative prognostic marker for recurrence in stage II-III CRC, providing surveillance lead times of 3–11 months over conventional methods. The landmark DYNAMIC trial demonstrated that ctDNA-guided adjuvant therapy safely reduced chemotherapy uses without compromising survival. By integrating ctDNA’s temporal “signal” with imaging’s spatial “localization,” clinicians can accelerate the detection of oligometastatic relapse, personalize surveillance, and refine treatment monitoring. This review synthesizes the evidence supporting this integrated approach, outlining the path toward a proactive, precision-based standard of care in postoperative CRC management, while also addressing the key challenges of standardization and clinical validation that must be overcome.

## 1 Introduction

Colorectal Cancer (CRC) represents a formidable global public health challenge. According to global cancer statistics, CRC is the third most diagnosed malignancy worldwide in both men (following lung and prostate) and women (following breast and lung) and remains a primary driver of cancer-related mortality (Simon, 2016; Zaki et al., 2022). Despite overall declines in cancer mortality, an alarming trend has emerged: the rapidly rising incidence of CRC in younger populations. Data indicate that CRC incidence among adults under 55 years of age is increasing by 1%–2% annually (Simon, 2016; O'Connell et al., 2004; Siegel et al., 2023). This epidemiological shift has had devastating consequences. Today, it has become the leading cause of cancer death within this age group (Bhandari et al., 2017).

For localized CRC, curative-intent surgical resection remains the primary treatment modality. However, the intent of a “curative” resection is frequently subverted by postoperative recurrence, which poses a substantial threat to long-term patient survival. The risk of recurrence is significant and pervasive across stages. Even among the earliest Stage I patients, recurrence rates are reported between 5.0% and 15.04%, indicating considerable heterogeneity even in early-stage disease (Nors et al., 2024; Gura and ya, 2019). For Stage II patients, the 5-year disease-free survival (DFS) is approximately 81.9%, with 5-year cumulative recurrence rates between 9.3% and 13% (Nors et al., 2024; Xiong et al., 2023; Böckelman et al., 2015). The prognosis is more severe for Stage III patients, where the 5-year DFS drops to 73.5%, signifying that over a quarter of these patients will experience disease relapse (Nors et al., 2024; Böckelman et al., 2015; Chen et al., 2021). Given these stark disparities, timely, affordable, and reliable prognosis and survival prediction are essential for precision clinical oncology. Accurate predictions pave the way for personalized risk and prognostic stratification, empowering clinicians to make informed treatment decisions and manage patient care effectively.

This high rate of relapse illuminates a central dilemma in current clinical practice: postoperative recurrence is not a *de novo* disease, but rather the clinical manifestation of pre-existing, occult micrometastatic disease that evades detection by conventional means—namely, Minimal Residual Disease (MRD) (Chakrabarti et al., 2022; Bork et al., 2014). The current gold standard for clinical risk stratification, the TNM staging system, relies predominantly on macroscopic assessment via conventional imaging (CT, MRI) and histopathology (Van Cutsem et al., 2016; García-Figueiras et al., 2018; Caruso et al., 2024). However, the high frequency of recurrence explicitly demonstrates the limitations of TNM staging in identifying patients harboring MRD; the resolution of these conventional tools is insufficient to detect disease burden at the microscopic level.

This limitation of TNM staging creates two critical clinical challenges: the “over-treatment” of patients who may already be cured by surgery alone (e.g., a subset of Stage II patients) (Habib et al., 2025), exposing them to the significant toxicities of adjuvant chemotherapy; and the “under-treatment” of patients harboring MRD who are otherwise classified as low-risk by conventional staging, providing a false sense of security (Yang et al., 2024). Consequently, a paradigm shift in clinical practice is urgently required. We transition beyond the current reliance on “anatomical detection” of macroscopic relapse and move toward the “molecular prediction” and “early functional detection” of occult disease. This mini review aims to explore this emerging paradigm: the integration of advanced imaging technologies and multi-omics (particularly liquid biopsies) to redefine CRC postoperative risk stratification and achieve truly individualized surveillance strategies.

## 2 Advanced imaging for postoperative surveillance: the role of radiomics

Standard-of-care postoperative surveillance relies heavily on scheduled computed tomography (CT) of the chest, abdomen, and pelvis, with magnetic resonance imaging (MRI) often reserved for pelvic surveillance in rectal cancer or problem-solving. These modalities, when interpreted conventionally by radiologists, function as anatomical tools. They are designed to detect macroscopic structural changes--such as new masses, enlarging lymph nodes, or organomegaly. This approach is inherently reactive; it identifies recurrence only after a significant tumor burden has been established, often failing to detect the micro-metastatic disease responsible for relapse during the window of curability.

### 2.1 Defining radiomics: mining the invisible

Radiomics involves the high-throughput extraction of large-scale quantitative features—such as texture, shape, and gray-level histograms—from standard medical images (CT, MRI, PET) using automated algorithms. This process converts medical images into a high-dimensional, mineable data space (Avery et al., 2022). The core philosophy underlying this approach is Radiogenomics, which posits that imaging phenotypes serve as macroscopic surrogates for microscopic pathophysiological changes.

Evidence suggests that radiomic features can non-invasively reflect tumor heterogeneity and genotype. For instance, specific textural features, such as entropy, have been strongly correlated with KRAS mutation status and Microsatellite Instability (MSI) (Porto-Álvarez et al., 2023). Studies by Hu et al. and Fan et al. have demonstrated that radiomic signatures can effectively discriminate between MSI-High and Microsatellite Stable (MSS) tumors, achieving an Area Under the Curve (AUC) of up to 0.908 (Li et al., 2024). This “virtual biopsy” capability enables pre-emptive risk stratification in the postoperative setting, overcoming the sampling bias inherent in traditional tissue biopsies (Ma et al., 2022).

### 2.2 Prognostic performance in clinical scenarios

A substantial body of evidence indicates that radiomic models exhibit superior accuracy in predicting postoperative recurrence and treatment response, effectively addressing the limitations of traditional TNM staging in resolving complex risk stratification scenarios.

#### 2.2.1 Colon cancer and CT-based models: precision risk stratification

In colon cancer surveillance, CT-based radiomics is primarily leveraged to resolve the prognostic uncertainty—often termed the “grey zone”—regarding recurrence risk in Stage II and III patients. In a landmark study addressing the preoperative prediction of lymph node metastasis, Huang et al. (JCO 2016) developed a radiomics nomogram based on portal venous phase CT. This model achieved a concordance index (C-index) of 0.778 in the validation cohort, significantly outperforming clinical staging alone and demonstrating the capacity of radiomics to capture microscopic invasive characteristics (Huang et al., 2016). Addressing the clinical dilemma of adjuvant chemotherapy allocation in Stage II patients, constructed a “Rad-score” model that successfully stratified patients into distinct risk categories. When integrated with clinical factors, this model predicted recurrence with an AUC of 0.872, providing a robust tool for identifying candidates who would genuinely benefit from adjuvant therapy (Fan et al., 2021). Furthermore, regarding the prediction of metachronous liver metastasis (MLM), developed a fusion model validated in a multicenter cohort. Achieving an AUC of 0.79, this model effectively identified primary tumors harboring a biological propensity for distant dissemination (Li et al., 2022). These advancements in CT-based radiomics for colon cancer have parallels in rectal cancer, where MRI-based radiomics plays a critical role in managing locally advanced disease.

#### 2.2.2 Rectal cancer and MRI-based models: surveillance of locally advanced disease

In Locally Advanced Rectal Cancer (LARC), radiomics based on multiparametric MRI (mpMRI) has emerged as a critical tool for predicting both the response to neoadjuvant chemoradiotherapy (nCRT) and long-term survival outcomes. The accurate prediction of Pathological Complete Response (pCR) is a prerequisite for organ-preservation strategies, such as “Watch and Wait.” Liu et al. extracted features from pre- and post-treatment MRI, achieving an exceptional AUC of 0.976 for pCR prediction. Similarly, reported that a radiomic classifier (AUC 0.93) significantly outperformed qualitative assessment by radiologists (Horvat et al., 2018). These findings were recently substantiated by a meta-analysis by Rai et al. (2025), which aggregated data from 35 studies to confirm a pooled AUC of 0.87 for MRI radiomics in predicting pCR, noting superior performance with MRI (AUC 0.90). Beyond response assessment, studies by Shin et al. (2022) have demonstrated that MRI radiomic signatures—particularly those derived from T2-weighted images—are independent predictors of disease-free survival (DFS), yielding C-indices between 0.77 and 0.82, which are superior to conventional clinical models. Moreover, emerging Deep Learning (DL) approaches have further elevated prognostic precision, with reported C-indices ranging from 0.82 to 0.94 (Shi et al., 2025).

### 2.3 Delta radiomics

Traditional radiomics typically analyzes medical images from a single time point (such as a baseline CT scan before treatment), while Delta radiomics focuses on the longitudinal assessment of quantitative image features, and analyze changes between distinct time points to quantify the dynamic evolution of tumor phenotypes in response to therapeutic intervention. In the context of Locally Advanced Rectal Cancer (LARC), Boldrini et al. demonstrated that early longitudinal changes in specific textural features during radiotherapy could predict pathological complete response (pCR) with high accuracy (Boldrini et al., 2019). Extending this analysis to the peritumoral environment, Chiloiro et al. (2023) reported that incorporating delta features from the mesorectum significantly enhanced the prediction of 2-year DFS (AUC 0.79), highlighting the critical prognostic relevance of the tumor microenvironment. Furthermore, in the setting of colorectal liver metastases, Giannini et al. (2022) established that Delta Radiomics outperforms standard RECIST criteria in evaluating chemotherapy response; their model achieved 93% accuracy in identifying non-responding lesions—compared to only 67% for RECIST—thereby providing critical evidence to avoid the continuation of ineffective therapeutic regimens.

In summary, advanced imaging—particularly radiomics—provides the essential spatial and risk context for surveillance, with studies reporting AUCs of 0.70–0.85 for preoperative and prognostic tasks. These performances, however, are influenced by methodological variability across studies, highlighting the need for standardization. While radiomics is thus a validated tool for risk stratification, it remains a prognostic rather than a real-time molecular detector. To identify recurrence before structural changes occur, a complementary, fluid-based approach is therefore required.

## 3 Multi-omics for molecular risk stratification in postoperative CRC surveillance

Postoperative surveillance of CRC faces persistent challenges due to the limitations of traditional TNM staging systems, which fail to recapitulate the profound molecular heterogeneity inherent to the disease. In such conditions, the “multi-omics” paradigm demonstrates unique value by integrating high-dimensional data across genomics, epigenomics, transcriptomics, proteomics, and microbiomics. Such distinct integration deciphers the complex biological mechanisms driving recurrence, enabling a refinement of risk stratification that transcends anatomical staging (Ullah et al., 2022; Liu Y. et al., 2025; Wang et al., 2024; Liu J. et al., 2025). This is where multi-omics, and liquid biopsy in particular, enters the surveillance paradigm.

### 3.1 Liquid biopsy and epigenetic synergies for MRD detection

The most transformative clinical application of this paradigm is the detection of Minimal Residual Disease (MRD) via liquid biopsy. Unlike static tissue biopsies, liquid biopsy serves as a dynamic tool for monitoring tumor burden through the analysis of circulating tumor DNA (ctDNA) (Vojjala et al., 2025). Recent evidence indicates that postoperative ctDNA status serves as a definitive predictor of outcome because ctDNA-positive patients face a significantly higher recurrence rate compared to their negative counterparts. Notably, this molecular detection often precedes radiographic evidence by several months (Potievskaya et al., 2025). This “window of opportunity” is a pivotal determinant for guiding adjuvant chemotherapy decisions, facilitating evidence-based treatment de-escalation for ctDNA-negative patients while justifying intensified regimens for high-risk, MRD-positive individuals (Zhang et al., 2025).

However, single-analyte approaches remain the main obstacles to maximizing sensitivity. Consequently, combinatorial regimens necessitate optimization to enhance detection accuracy. For instance, genomic profiling is increasingly augmented by epigenomics through the analysis of methylated DNA markers (MDMs). Since aberrant DNA methylation is a key driver of CRC progression, MDMs serve as highly specific biomarkers (Zhu et al., 2023; Ogaard et al., 2024). Studies utilizing ultra-deep sequencing have illustrated that the combined analysis of ctDNA mutations and methylation patterns yields superior sensitivity compared to single-analyte strategies, a synergistic effect that is particularly valuable in high-risk cohorts, such as patients with colorectal peritoneal metastases, where a postoperative “dual-negative” status correlates with significantly improved overall survival (Chen et al., 2025).

However, the reported lead time and sensitivity of ctDNA are subject to pre-analytical and analytical variability, influenced by factors such as sequencing depth, panel design, and bioinformatic pipelines (Tao et al., 2024; Ogaard et al., 2022). Clinical implementation also faces challenges including cost, turnaround time, and the need to distinguish tumor-derived signals from clonal hematopoiesis (Ogaard et al., 2022; Huang et al., 2025). Acknowledging these variables is essential for interpreting study results and guiding the reliable integration of ctDNA into routine surveillance.

### 3.2 Multidimensional profiling of the tumor microenvironment

Beyond the circulation, a broader spectrum of omics technologies provides complementary insights into the tumor microenvironment (TME). The gut microbiome has emerged as a robust independent prognostic indicator. Machine learning analyses have revealed that tissue bacterial community composition can outperform traditional transcriptomic markers in predicting overall survival (AUC 0.755 vs. 0.702) (Yang et al., 2022), and specific dysbiotic patterns are consistently linked to recurrence (Liu B. et al., 2025). Parallel advancements in proteomics have identified functional markers such as CAVIN1 in aggressive mesenchymal subtypes (Martinez-Val et al., 2025), while metabolomics and lipidomics have elucidated distinct lipid metabolic signatures that allow for precise survival stratification (Fu et al., 2024). Additionally, transcriptomic signatures based on relative expression ordering provide stable prognostic features for predicting chemotherapy response (Tong et al., 2016). The true power of these diverse omics layers, however, lies not in their isolation but in their integration, which allows for a more complete resolution of tumor heterogeneity.

### 3.3 Multi-omics integration and resolution of tumor heterogeneity

The multi-omics paradigm is fully realized by synthesizing heterogeneous, multi-layered datasets into a high-resolution, functional portrait of the patient’s disease state—a process that demands advanced computational frameworks to manage its high-dimensional complexity (Mandala, 2025). This integrative strategy harnesses cross-platform synergy to construct robust prognostic models, exemplified by the Multi-omics Clustering and Machine Learning Scoring (MCMLS) model, which fuses transcriptomics, epigenomics, genomics, and microbiome data to significantly outperform established signatures derived from single-omics data (Wang et al., 2025). To further refine this molecular landscape, incorporating single-cell and spatial transcriptomics elevates surveillance to the cellular level, effectively resolving tumor heterogeneity and immune evasion mechanisms that bulk sequencing often obscures (Wen et al., 2025; Luan et al., 2025).

In summary, multi-omics delivers a sensitive, functional portrait of dynamic disease biology. To translate this molecular ‘signal’ into actionable clinical guidance, it must be paired with the spatial ‘localization’ capability of imaging—a synergy that defines the next frontier in precision surveillance. A comparative overview of advanced imaging, multi-omics, and integrated fusion paradigms for postoperative colorectal cancer surveillance is summarized in Table 1.

**TABLE 1:** Comparative overview of advanced imaging, multi-omics, and integrated fusion paradigms.

Surveillance paradigm	Key modalities	Biological focus	Clinical advantages	Limitations	Ref.
Advanced imaging	CT radiomics	Tumor heterogeneity; Genotype prediction; Micro-metastasis	Adjuvant therapy Decision; Occult Metastasis Predicion	Acquisition variability; Anatomical Noise	Huang et al. (2016), Fan et al. (2021), Li et al. (2022)
	MRI radiomics	Tissue viability vs. fibrosis; Tumor Microenvironment	pCR Prediction; Survival Prediction	Standardization	Horvat et al. (2018), Rai et al. (2025), Shin et al. (2022)
Multi-omics	Liquid biopsy	MRD Detection and synergy: Dynamic tumor burden monitoring; epigenetic markers augment genomic specificity	Window of opportunity: Recurrence prediction preceding radiography; guidance for chemotherapy modulation; superior survival correlation	Spatially indeterminate: Lack of spatial resolution for localization; limited single-analyte sensitivity in low-volume disease	Vojjala et al. (2025), Potievskaya et al. (2025), Zhang et al. (2025), Zhu et al. (2023), Ogaard et al. (2024), Chen et al. (2025)
	Other omics	TME and functional profiling: Elucidation of TME dysbiosis, metabolic reprogramming, and functional protein markers	Independent prognosis: Superior prognostic accuracy over transcriptomics; precise metabolic risk stratification	Standardization gaps: High data dimensionality; lack of standardized sampling and analytical protocols	Yang et al. (2022), Liu et al. (2025c), Martinez-Val et al. (2025), Fu et al. (2024), Tong et al. (2016)
	Multi-omics integration	Resolution of heterogeneity: Synthesis of multi-layered datasets; resolution of cellular heterogeneity and immune evasion mechanisms	Functional portrait: Comprehensive functional disease profiling; superior prognostic performance over single-omics signatures	Technical complexity: Prohibitive computational demands and sequencing costs limiting clinical accessibility	Mandala (2025), Wang et al. (2025), Wen et al. (2025), Luan et al. (2025)
Integrated fusion	Sequential-trigger model	Diagnostic optimization: Utilization of sensitive molecular signals as triggers for specific reflex imaging	Efficiency and safety: Reduced diagnostic delays; minimization of unnecessary radiation; targeted localization of occult metastases	Sequential dependency: Efficacy strictly contingent upon initial molecular trigger sensitivity	Ogaard et al. (2022), Reinert et al. (2022), Krishnan and Mukherjee (2025)
	Radiogenomics	Virtual biopsy: Quantification of imaging phenotypes as macroscopic surrogates for genomic alterations	Non-Invasive monitoring: Continuous tracking of clonal evolution; assessment of chemotherapy response without tissue sampling	Data heterogeneity: Sensitivity to scanner batch effects; requirement for rigorous standardization for generalization	Elahi et al. (2025), Granata et al. (2023)
	Deep fusion of imaging and Multi-omics	Morpho-Molecular Synergy: Deep learning synthesis of spatial and functional data into a Digital Twin for adaptive management	Precision and adaptation: Identification of high-risk MRD-negative subsets; facilitation of risk-stratified adaptive follow-up	Opacity and validation: Algorithmic opacity hindering clinical trust; lack of large-scale prospective validation	Loeffler et al. (2025), Tsai et al. (2023), Cicalini et al. (2024), Huang et al. (2023), Qiu et al. (2025), Xiong et al. (2025)

## 4 Precision surveillance through integrated advanced imaging and multi-omics

**FIGURE 1:**
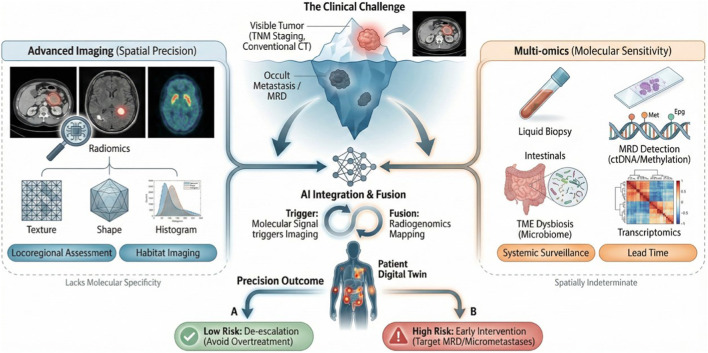
From “Blind Spots” to “Holographic lnsight”: lntegrating Advanced lmaging and Multi-omics for Precision CRC Management.

Postoperative surveillance for CRC is gradually moving into the limelight of research as it transitions from reactive monitoring toward a unified, predictive framework. Current standard-of-care protocols frequently generate a diagnostic blind spot because advanced imaging provides spatial context but lacks specificity for molecular recurrence, while multi-omics offers high sensitivity for residual disease yet remains spatially indeterminate. For instance, ctDNA sensitivity often remains limited compared to functional imaging in localizing low-volume tumor burden such as early peritoneal carcinomatosis (Dawood et al., 2023), whereas traditional anatomical imaging struggles to distinguish viable tumor foci from post-surgical scarring (Tao et al., 2024). The emerging surveillance paradigm necessitates the synergistic integration of these orthogonal data streams to construct a dynamic biological profile of the CRC patient. This emerging paradigm, which bridges molecular sensitivity with spatial localization, is schematically illustrated in Figure 1.

### 4.1 The sequential-trigger model for diagnostic optimization

Integrating these complementary data streams—imaging and multi-omics—into clinical workflow presents a practical challenge. The “sequential-trigger” model represents the most immediate clinical realization of this integration, designed to resolve ambiguity during routine follow-up. Indeterminate findings on postoperative CT scans occur frequently and traditionally necessitate a “watch-and-wait” approach that results in diagnostic delays (Ogaard et al., 2022). Integrating molecular biomarkers into this workflow fundamentally alters clinical decision-making. Evidence suggests that a positive multi-omics signal acts as a definitive trigger for reflex imaging using MRI or PET-CT to localize occult metastases (Reinert et al., 2022). This strategy represents a new breakthrough direction that not only targets recurrence more effectively but also potentially reduces unnecessary radiation exposure from equivocal scans by up to 40% (Krishnan and Mukherjee, 2025).

### 4.2 Radiogenomics as a non-invasive biological surrogate

Moving beyond sequential applications, radiogenomics provides the biological rationale for continuous, non-invasive monitoring. This discipline posits that quantitative imaging phenotypes serve as macroscopic surrogates for multi-omics profiles. Research validates that radiomic textures can non-invasively predict key CRC driver mutations such as KRAS and BRAF, functioning as a “virtual biopsy” to track clonal evolution (Elahi et al., 2025). Crucially for longitudinal surveillance, “Delta-Radiomics” analyzes the temporal evolution of image features and has proven effective in monitoring responses to adjuvant chemotherapy (Granata et al., 2023). This establishes anatomical imaging as a dynamic repository of latent genomic information, justifying its computational fusion with fluid biomarkers.

### 4.3 AI-driven deep fusion of imaging and multi-omics landscapes

The most advanced form of integration moves beyond sequential or correlative models toward a deep, AI-driven fusion, creating a system where each modality compensates for the other’s limitations The HIBRID model exemplifies this morpho-molecular synergy by fusing deep learning risk scores from histology with ctDNA status (Loeffler et al., 2025). The study illustrated that this model identified a subgroup of MRD-negative patients who exhibited high-risk morphological features and derived significant survival benefit from adjuvant chemotherapy, with 24-month disease-free survival improving from 69% to 84%. Similarly, deep learning frameworks like MOMA bridge tissue morphology with broad multi-omics aberrations to refine prognosis (Tsai et al., 2023).

Finally, this paradigm expands into metabolic and transcriptomic dimensions. Novel “radiometabolomics” frameworks combining MRI radiomics with untargeted metabolomics have demonstrated superior accuracy in predicting treatment responses for organ-preservation strategies (Cicalini et al., 2024). Furthermore, integrating PET imaging with proteomics has successfully elucidated mechanisms of glucose metabolic reprogramming in colorectal liver metastases (Huang et al., 2023; Qiu et al., 2025). Additionally, linking CT radiomics with long non-coding RNA signatures enables the continuous monitoring of molecular subtypes (Xiong et al., 2025). Collectively, these advancements shift postoperative surveillance from a static schedule to a dynamic, predictive strategy. By fusing spatial precision with molecular sensitivity, this paradigm constructs a “digital twin” of the patient’s disease status.

## 5 Challenges and future directions

While the integration of advanced imaging and multi-omics offers a transformative blueprint for CRC surveillance, bridging the gap between computational proof-of-concept and clinical utility remains a formidable challenge. Realizing this new paradigm necessitates navigating a landscape of technical heterogeneity, algorithmic opacity, and the rigorous demands of prospective validation.

### 5.1 Data heterogeneity and standardization protocols

The most significant technical impediment is data heterogeneity. Radiomic features are notoriously sensitive to batch effects caused by variations in scanner parameters, and multi-omics data similarly suffer from platform-specific variations. Consequently, AI models trained in single centers frequently fail to generalize externally as they learn institutional noise rather than true pathology (Cicalini et al., 2024; Huang et al., 2025). In the future, implementation strategies need to be optimized through harmonization protocols and adherence to universal standards like the Image Biomarker Standardisation Initiative (IBSI). This standardization is essential to ensure models represent reproducible clinical tools rather than localized experiments (Huang et al., 2025).

### 5.2 Algorithmic opacity and explainable artificial intelligence

Beyond technical stability, the “black box” nature of deep learning remains a main obstacle to transformation. In the high-stakes context of surveillance, clinicians are hesitant to rely on opaque algorithmic outputs for decisions regarding toxic adjuvant therapy. Future research must pivot toward Explainable AI (XAI) to visualize the “decision path” by highlighting specific intratumoral textures or molecular pathways. This approach aims to transition from a “black box” to a transparent “glass box” to foster the confidence necessary for routine adoption (Tsai et al., 2023).

### 5.3 Prospective validation and adaptive management strategies

Furthermore, the current evidence base relies heavily on retrospective studies prone to selection bias. Validating clinical utility requires large-scale, multi-center prospective trials to ensure robustness across diverse populations (Huang et al., 2025). Establishing such collaborative networks is essential to overcome data silos. Ultimately, overcoming these barriers will evolve surveillance into an active and adaptive management loop. By integrating predictive models into a dynamic patient “Digital Twin,” clinicians can tailor follow-up intensity by safely de-escalating for concordant low-risk profiles while intensifying for discordant high-risk signals (Loeffler et al., 2025). This strategy, by reshaping the surveillance landscape, will be an attractive strategy to break the bottlenecks in postoperative management and transform CRC care into a personalized, data-driven science.

## 6 Conclusion

The transition from reactive anatomical monitoring to proactive molecular prediction represents a pivotal evolution in postoperative colorectal cancer surveillance. As highlighted in this review, traditional TNM staging and conventional imaging often fail to capture the biological complexity of Minimal Residual Disease (MRD), leading to missed opportunities for early intervention. The integration of advanced imaging and multi-omics offers a transformative solution to this challenge. Radiomics provides crucial spatial context and non-invasive risk stratification, while liquid biopsy—particularly ctDNA—delivers unparalleled sensitivity for detecting molecular recurrence with significant lead times.

By synergizing these orthogonal data streams, clinicians can overcome the diagnostic blind spots inherent to single-modality approaches, moving towards a “morpho-molecular” precision framework. However, the clinical realization of this paradigm requires rigorous efforts to address data heterogeneity, standardize acquisition protocols, and validate AI-driven models through large-scale prospective trials. Ultimately, bridging these gaps will pave the way for dynamic, personalized surveillance strategies—akin to a patient “Digital Twin”—that optimize survival outcomes while minimizing unnecessary treatment toxicity.
